# Quantitative evaluation of correlation between lumbosacral lordosis and pelvic incidence in standing position among asymptomatic Asian adults: a prospective study

**DOI:** 10.1038/s41598-022-21840-x

**Published:** 2022-11-08

**Authors:** Jie-Ren Mi Le, Kuang-Ting Yeh, Chih-Wei Chen, Fu-Shan Jaw, Shu-Hua Yang, Wen-Tien Wu

**Affiliations:** 1 Department of Biomedical Engineering, College of Medicine and College of Engineering, National Taiwan University, Taipei, 10617 Taiwan; 2 Department of Orthopedics, Hualien Tzu Chi Hospital, Buddhist Tzu Chi Medical Foundation, Hualien, 970473 Taiwan; 3 School of Medicine, Tzu Chi University, Hualien, 970374 Taiwan; 4 Department of Orthopedics, National Taiwan University College of Medicine and National Taiwan University Hospital, Taipei, 100225 Taiwan

**Keywords:** Anatomy, Health care, Medical research

## Abstract

The determination of lumbopelvic alignment is essential for planning adult spinal deformity surgery and for ensuring favorable surgical outcomes. This prospective study investigated the correlation between the lumbar section of lumbar spine lordosis and increasing pelvic incidence in 324 Asian adults with a mean age of 55 ± 13 years (range: 20–80 years), comprising 115 male and 209 female volunteers. Participants were divided into three groups based on pelvic incidence (G1, G2, and G3 had pelvic incidence of < 45°, 45–55°, and ≥ 55°, respectively). We determined that distal and proximal lumbar lordosis contributed differentially to the increase in pelvic incidence, whereas the lordosis ratio of the L3–L4 and L4–L5 segments mostly remained constant. The mean contribution ratio of the segmental lordosis from L1 to S1 was as follows: L1–L2, 2.3%; L2–L3, 11.7%; L3–L4, 18.1%; L4–L5, 25.2%; and L5–S1, 42.7%. Pelvic incidence had a stronger correlation with proximal lumbar lordosis than did distal lumbar lordosis. The ratios of proximal lumbar lordosis to distal lumbar lordosis were 37.8% in G1, 45.8% in G2, and 55.9% in G3. These findings serve as a reference for future lumbar spine correction or fusion surgery for Asian adults.

## Introduction

Lumbopelvic alignment is critical given its influence on spinal biomechanics^1^, health-related quality of life^2^, and surgical outcomes^3–5^. Postural changes, surgical implants, and any pathological factors that alter spinal balance can lead to sagittal misalignment and the subsequent effectuation of a compensatory mechanism^6^. Therefore, sagittal alignment is vital for preserving spinal function, especially for the lumbar region.

The lumbar region can withstand various contact forces and is the main weight-bearing region of the spine^1,7–9^. In lumbar hypolordosis, the vertical force on the vertebral endplate is greater than the parallel force. Spinal degeneration may cause the progression of this condition^1,10^. Conversely, in lumbar hyperlordosis, the force vector parallel to the endplate is higher than the perpendicular vector. An increased shearing force increases the risks of posterior facet arthritis and spondylolisthesis^11,12^.

Lumbar lordosis is the inward curve of the spine formed by the wedging angle of the vertebral bodies and discs^13–15^. Each lumbar segment variously contributes to lordosis. The lower segment has a high lordosis angle^14,15^. L1 contributes − 4 to 4% and L5 contributes 40% to 52% to the morphology of lordosis^14–17^. Because the lumbar region has a high range of movement, lumbar lordosis changes with posture. Studies have reported that lumbar lordosis in asymptomatic adults (asymptomatic of neck pain or back pain) is greater in the standing position than in the supine position and greater in the supine position than in the sitting position. Specifically, in the standing position, lordosis is approximately 8° greater than in the supine position and 30° greater than in the sitting position^18,19^.

Pelvic incidence is a key indicator of pelvic health and is associated with sagittal alignment. Studies have indicated that pelvic incidence varies from 30 to 80°^15,20,21^ and differs across ethnicities^22,23^. Pelvic incidence is mostly constant in asymptomatic adults and is significantly correlated with lumbar lordosis^20,24^. The researchers have explored other parameters that affect pelvic incidence in the last decade. Arand et al. determined that pelvic incidence and acetabular anteversion exhibited a positive correlation^25^. Kobayashi et al. discovered that in patients with hip osteoarthritis without hyperlordosis, standing pelvic incidence was significantly associated with lumbar lordosis^26^, which corresponds to the finding that high pelvic incidence with hyperlordosis is associated with a more sagittal orientation of the lumbar facet joint in patients with facet joint arthritis^27,28^.

Various conclusions have been drawn from studies examining the association between pelvic morphology and the lumbar segments contributing to lumbar lordosis. In 2018, on the basis of anatomic parameters, Pesenti et al. demonstrated that only proximal lumbar lordosis varied according to pelvic incidence^17^. In 2021, Li et al. demonstrated that both proximal lumbar lordosis and distal lumbar lordosis were correlated with pelvic incidence, and the correlation of proximal lumbar lordosis with pelvic incidence was stronger^29^. Because accurate realignment for lumbar lordosis is still not well understood, a comprehensive investigation of the association between pelvic incidence and lumbar lordosis in the Asian population is necessary. This study included asymptomatic Asian adults, divided participants into three groups according to pelvic incidence, and investigated the variation of lumbar lordosis according to pelvic morphological and lumbar segmental aspects among the three groups. The information should be useful for planning spinal realignment surgery.

## Results

The study population comprised 115 men and 209 women, and their mean age was 55 years (range: 20–80 years). The mean value of lumbar lordosis was 50.3° ± 11.9° (Table 1). The mean values of proximal lumbar lordosis and distal lumbar lordosis were 16.1° ± 8.3° and 34.2° ± 8.8°, respectively, and accounted for 32% and 68% of total lumbar lordosis, respectively. The participants were divided into the following three groups on the basis of pelvic incidence: G1 was the high pelvic incidence group, comprising those with a pelvic incidence of < 45°; G2 was the moderate pelvic incidence group, comprising those with a pelvic incidence of 45–55°; and G3 was the low pelvic incidence group, comprising those with a pelvic incidence of > 55°. Mean values for proximal lumbar lordosis and distal lumbar lordosis among the three groups differed significantly (proximal lumbar lordosis: G1, 11.6° ± 6.7° vs. G2, 16° ± 7.7° vs. G3, 20.7° ± 8°, and distal lumbar lordosis: G1, 30.7° ± 8.1° vs. G2, 34.9° ± 7.9° vs. G3, 37° ± 9.3°; *P* < 0.001). Both pelvic tilt and sacral slope increased with pelvic incidence and exhibited strong correlations (pelvic tilt: G1, 8.6° ± 6.8° vs. G2, 12.3° ± 6.9° vs. G3, 19.5° ± 6.5°, and sacral slope: G1, 29.7° ± 6.8° vs. G2, 37.3° ± 6.9° vs. G3, 43.8° ± 6.8°; *P* < 0.001). G3 (high pelvic incidence) had the highest values for lumbar lordosis, proximal lumbar lordosis, distal lumbar lordosis, pelvic tilt, and sacral slope, whereas G1 (low pelvic incidence) had the lowest values for the aforementioned parameters. The lumbar apex shifted proximally from G1 to G3 (G1, center of L4 vs. G2, top L4 vs. G3, L3–L4; *P* < 0.001). Parameters including thoracic kyphosis, the difference between pelvic incidence and lumbar lordosis, and the C7–S1 sagittal vertical axis did not significantly differ among the three groups.

**Table 1:** Radiographic analysis for different pelvic incidence groups. BMI: bone mass index; DL: distal lumbar lordosis; G1: pelvic incidence < 45°; G2: pelvic incidence between 45°–55°; G3: pelvic incidence > 55°; LL: lumbar lordosis; PI-LL: the difference between pelvic incidence and lumbar lordosis; PL: proximal lumbar lordosis; PT: pelvic tilt; SS: sacral slope; SVA: C7-S1 sagittal vertical axis; TK: thoracic kyphosis.

Item	Difference pelvic incidence groups			Total 50.5 ± 11.4	ANOVA *P* value	Post-hoc
	G1 (*n* = 105) 38.3 ± 4.8	G2 (*n* = 111) 49.6 ± 2.8	G3 (*n* = 108) 63.3 ± 7.0			
Age	56.8 ± 14.3	54.2 ± 12.1	54.3 ± 13.1	55.1 ± 13.2	0.255	
BMI	25.2 ± 3.7	25.8 ± 3.8	24.9 ± 4.7	25.3 ± 4.1	0.259	
TK	− 35.2 ± 11.8	− 34.4 ± 15.3	− 33.5 ± 13.1	− 34.4 ± 13.5	0.670	
LL	42.2 ± 9.4	50.8 ± 10.8	57.7 ± 10.0	50.3 ± 11.9	< 0.001*	G1 < G2 < G3
PL	11.6 ± 6.7	16.0 ± 7.7	20.7 ± 8.0	16.1 ± 8.3	< 0.001*	G1 < G2 < G3
DL	30.7 ± 8.1	34.9 ± 7.9	37.0 ± 9.3	34.2 ± 8.8	< 0.001*	G1 < G2 < G3
PT	8.6 ± 6.8	12.3 ± 6.9	19.5 ± 6.5	13.5 ± 8.1	< 0.001*	G1 < G2 < G3
SS	29.7 ± 6.8	37.3 ± 6.9	43.8 ± 6.8	37.0 ± 8.9	< 0.001*	G1 < G2 < G3
PI-LL	8.3 ± 6.0	8.4 ± 6.4	9.2 ± 7.6	8.6 ± 6.7	0.526	
SVA	28.0 ± 23.6	33.5 ± 26.6	28.7 ± 24.4	30.1 ± 25.0	0.211	
Apex	Center of L4 ± 1 vertebrae	Top L4 ± 1 vertebrae	L3–L4 ± 1 vertebrae	Top L4 ± 1 vertebrae	< 0.001*	G1 > G2 > G3

Data are presented as frequency or mean ± SD.

*P < 0.05 was considered statistically significant.

**Figure 1:**
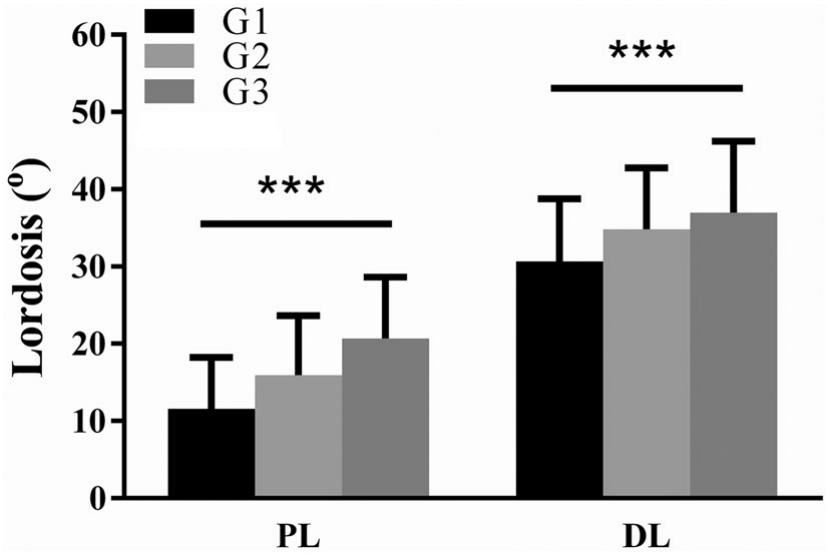
Bar chart of values of proximal lumbar lordosis and distal lumbar lordosis in the three pelvic incidence groups.

The mean value of pelvic incidence was 50.5° ± 11.4° (95% confidence interval: 49.3°–51.8°). Proximal lumbar lordosis and distal lumbar lordosis were the highest in G3 and the lowest in G1. Proximal lumbar lordosis values were smaller than distal lumbar lordosis values among the three groups (Fig. 1). The values of proximal lumbar lordosis and distal lumbar lordosis exhibited positive correlations with pelvic incidence (proximal lumbar lordosis: *r* = 0.491, *P* < 0.001; distal lumbar lordosis: *r* = 0.286, *P* < 0.001; Fig. 1).

**Figure 2:**
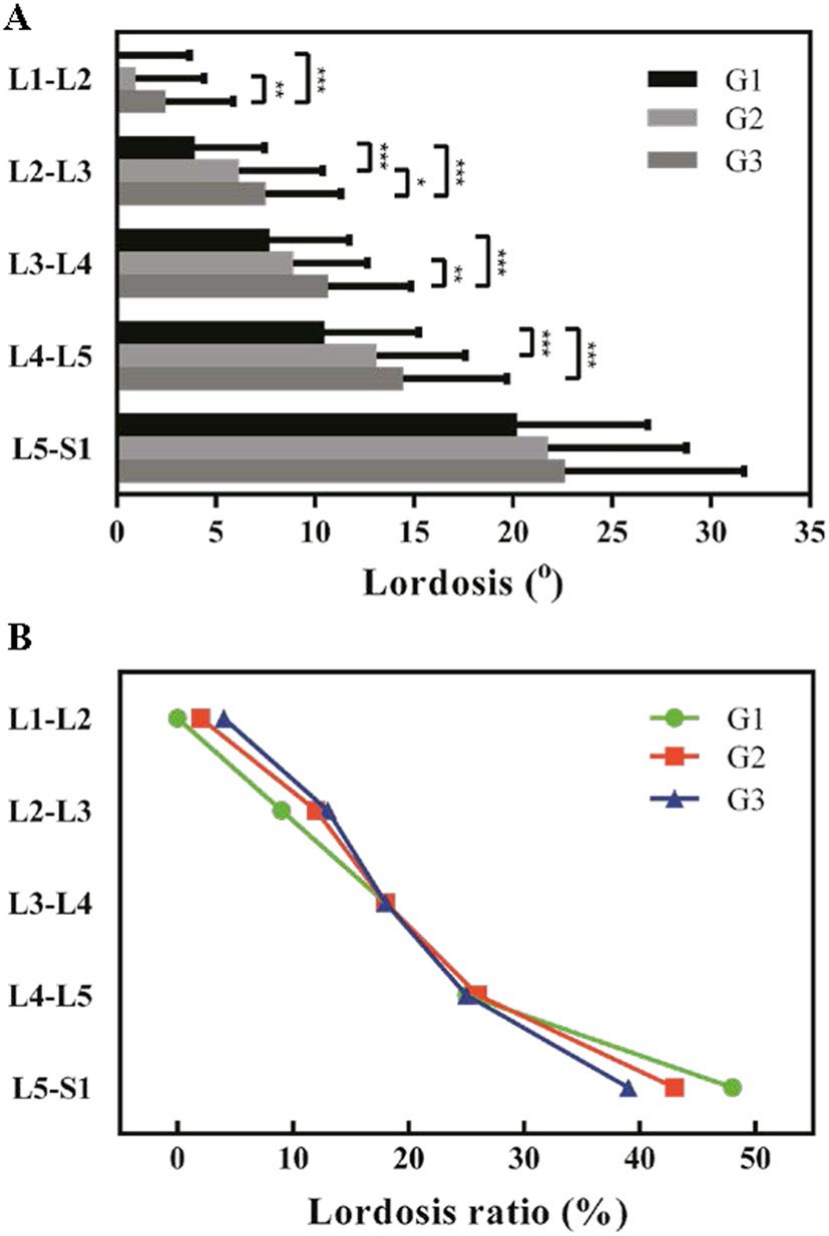
(**A**) Segmental lordosis of pelvic incidence groups in terms of degree. (**B**) Segmental lordosis of pelvic incidence groups in terms of ratio.

**Figure 3:**
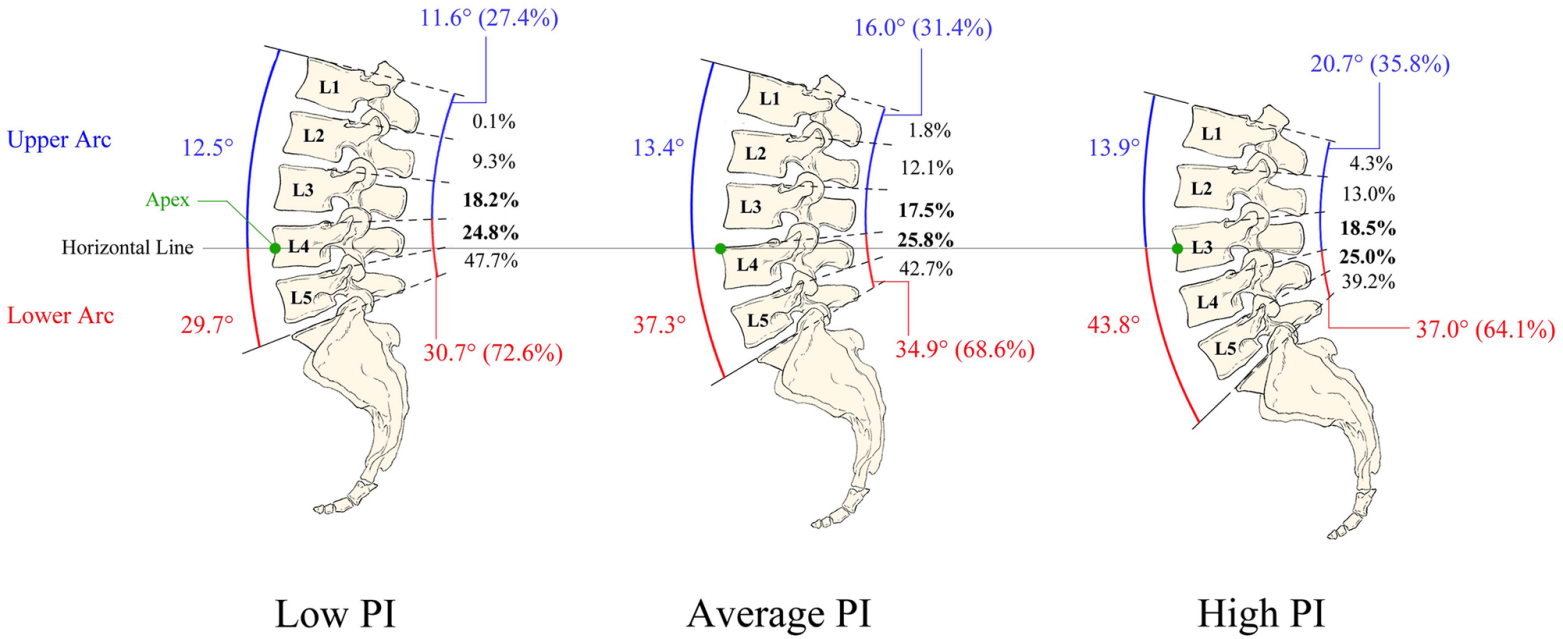
Correlation between pelvic morphology and lumbar spine. The angle between the horizontal line of the apex and the superior endplate of S1 (lower arc) is equal to the sacral slope, and the angle between the superior endplate of L1 and the horizontal line of the apex (upper arc) was obtained through subtracting the sacral slope from the lumbar lordosis angle.

The mean contribution percentages of segmental lordosis from L1 to S1 were as follows: L1–L2, 2.3%; L2–L3, 11.7%; L3–L4, 18.1%; L4–L5, 25.2%; and L5–S1, 42.7% (Table 2). The mean contribution value of each segment to lordosis was the lowest in G1 and the highest in G3. Furthermore, L1–L2 had the smallest mean value and L5–S1 had the largest mean value among the three groups (Fig. 2A). Ratio analysis revealed several differences. In G3, L1–L2 and L2–L3 exhibited a higher ratio of segmental lordosis than the same segments in G1, and the ratio of segmental lordosis at the L5–S1 segment was lower in G3 than in G1 (Fig. 2B). In addition, the lordosis ratios of the L3–L4 and L4–L5 segments, which were approximately 18% and 25%, respectively, were mostly constant among the groups. Examination of the profile of the lumbar spine revealed that the upper arc of lumbar lordosis was approximately 13° in the three groups (Fig. 3), with no significant difference observed among the groups (G1, 12.5° ± 6.2° vs. G2, 13.4° ± 6.8° vs. G3, 13.9° ± 7.1°; *P* = 0.411). The lower arc was strongly and positively correlated with pelvic incidence (the lower arc is equal to the sacral slope; G1, 29.7° ± 6.8° vs. G2, 37.3° ± 6.9° vs. G3, 43.8° ± 6.8°; *P* < 0.001).

**Table 2:** Segmental lumbar spine lordosis for the pelvic incidence groups. G1: pelvic incidence < 45°; G2: pelvic incidence between 45° and 55°; G3: pelvic incidence > 55°.

Segment	Difference pelvic incidence groups						Total	
	G1 (*n* = 105) 38.3 ± 4.8	Ratio	G2 (*n* = 111) 49.6 ± 2.8	Ratio	G3 (*n* = 108) 63.3 ± 7.1	Ratio	*n* = 324 50.5 ± 11.4	Ratio
L1–L2	0.0 ± 3.6	0.1%	0.9 ± 3.5	1.8%	2.5 ± 3.4	4.3%	1.2 ± 3.6	2.3%
L2–L3	3.9 ± 3.5	9.3%	6.2 ± 4.2	12.1%	7.5 ± 3.8	13.0%	5.9 ± 4.1	11.7%
L3–L4	7.7 ± 4.0	18.2%	8.9 ± 3.7	17.5%	10.7 ± 4.2	18.5%	9.1 ± 4.2	18.1%
L4–L5	10.5 ± 4.7	24.8%	13.1 ± 4.5	25.8%	14.4 ± 5.3	25.0%	12.7 ± 5.1	25.2%
L5–S1	20.2 ± 6.6	47.7%	21.8 ± 7.0	42.7%	22.6 ± 9.1	39.2%	21.6 ± 7.7	42.7%

## Discussion

Sagittal lumbar alignment is based on pelvic morphology, and this knowledge is helpful for planning surgeries to correct adult spinal deformities. Sagittal realignment surgeries have been performed on patients with degenerative spondylolisthesis, degenerative thoracolumbar kyphosis, ankylosing spondylitis–related kyphotic deformity, and posttraumatic thoracolumbar kyphotic deformity for functional recovery^3,30–32^. The back pain and spinal function of patients with spondylolisthesis or adult spinal deformity can be improved through the stabilization of their spinal structure and the surgical realignment of their global sagittal balance under the restoration of standing lumbar lordosis^33,34^. Because lumbar lordosis and pelvic parameters are positively correlated, carefully exploring the relationship between the lumbar sections contributing to lumbar lordosis and the different ranges of pelvic incidence is crucial.

The mean values of lumbar lordosis and pelvic incidence obtained in this study are similar to those in studies conducted in Asian populations^35,36^ but lower than those in Caucasian populations^13,17^. In the present study, proximal lumbar lordosis (32%) accounted for a lower proportion of total lumbar lordosis than did distal lumbar lordosis (68%). The value of distal lumbar lordosis obtained in this study was 4%–6% higher than the values previously reported in Caucasian populations^16,17^. In the present study, the normal distribution of pelvic incidence had a mean value of 50.5°, which was 4° lower than that in asymptomatic Caucasian populations^13,17^. To explore the differences in certain radiologic parameters, we divided our participants into three groups according to pelvic incidence (i.e., G1: < 45°, G2: 45°–55°, and G3: > 55°).

In this study, the ratios of proximal lumbar lordosis to distal lumbar lordosis were 37.8% in G1 (low pelvic incidence), 45.8% in G2 (moderate pelvic incidence), and 55.9% in G3 (high pelvic incidence). For proximal lumbar lordosis, the percentage increase between G1 and G2 and between G2 and G3 was 37.9% and 29.4%, respectively, but was only 13.7% (G1 to G2) and 6% (G2 to G3) for distal lumbar lordosis. Although proximal lumbar lordosis accounted for a smaller proportion of total lumbar lordosis than did distal lumbar lordosis, proximal lumbar lordosis had a greater percentage increase than distal lumbar lordosis from the low to the high pelvic incidence groups. These findings indicated that proximal lumbar lordosis and distal lumbar lordosis were simultaneously affected by the pelvic incidence value and that, between the groups, the variation of proximal lumbar lordosis was higher than that of distal lumbar lordosis. The implication for surgical planning is that even a minor angle adjustment can improve a patient’s sagittal alignment and prevent pathological conditions such as degeneration, facet joint arthritis, and spondylolisthesis caused by hypolordosis and hyperlordosis.

We determined that the lordosis ratios of the L3–L4 and L4–L5 segments were mostly constant among the groups, which implies that the lordosis ratios of these two segments are not affected by pelvic incidence. Figure 1 presents the segmental lordosis tendency among the three pelvic incidence groups. Above the L3–L4 segment, G3 exhibited the highest ratio among the groups. Conversely, G3 had the lowest ratio at the L5–S1 segment. This result implies that the point of intersection has a critical role in maintaining equilibrium between the upper and lower segments in biomechanics. The proportion of segmental lordosis at L1–L5 was lower than that reported in other studies (within 2%), whereas the proportion of segmental lordosis of the L5–S1 segment was 3%–8% higher than that in Caucasian populations^15,17^. Thus, our findings suggest that compared with Caucasian populations, the lumbar curvature of Asian people is slightly lower at the L1–L5 segments and significantly higher at the L5–S1 segments.

By applying a horizontal line at the apex, we observed the variation in the positions of the superior endplates of L1 and S1. The upper arc mostly remained constant (approximately 13°), whereas the lower arc exhibited high variability with pelvic incidence. Our results indicate that the superior endplate of L1 is relatively motionless, whereas the sacral endplate moves backward with an increase in pelvic incidence. This observation indicates that the increase of lumbar lordosis is mainly attributable to the increase of the backward shift of the sacral endplate. This not only corresponds to the finding that the lumbar apex shifts proximally with pelvic incidence but also explains the high correlation between pelvic incidence and lumbar lordosis.

This study has some limitations. First, it mainly focused on lumbopelvic alignment. Future studies should investigate the influence of the lower limbs and the morphology of the cervical and thoracic spine. Second, this was a population-specific study, and the results may not be applicable to non-Asian ethnicities. Further studies of different populations can help clinicians provide patient-specific treatment. Despite these limitations, the results indicate that ethnicity is associated with lumbar lordosis through the varying contributions of different lumbar sections. When making decisions related to lumbar curvature correction, surgeons must account for segmental lordosis to provide more patient-specific treatment. In addition to spinal parameters, parameters related to the lower extremities^33^ and paraspinal muscle^34^ are involved in spinal function and sagittal alignment. For example, the parameter of head-to-feet sagittal alignment can be analyzed in future studies. Despite the aforementioned limitations, the quantitative results of this study demonstrated an association between the lumbar sections of lumbar lordosis and pelvic incidence in the Asian population.

## Conclusion

This quantitative study revealed correlations between sections of lumbar lordosis and increased pelvic incidence in Asian adults. Both proximal lumbar lordosis and distal lumbar lordosis were correlated with pelvic incidence. Proximal lumbar lordosis exhibited a higher correlation with pelvic incidence than did distal lumbar lordosis. An increased pelvic incidence induces greater lumbar lordosis for maintaining balance. The lumbar apex was more proximal in the high pelvic incidence group than in the low pelvic incidence group. These findings may be used as a reference for future lumbar spine correction or fusion surgery.

## Materials and methods

**Figure 4:**
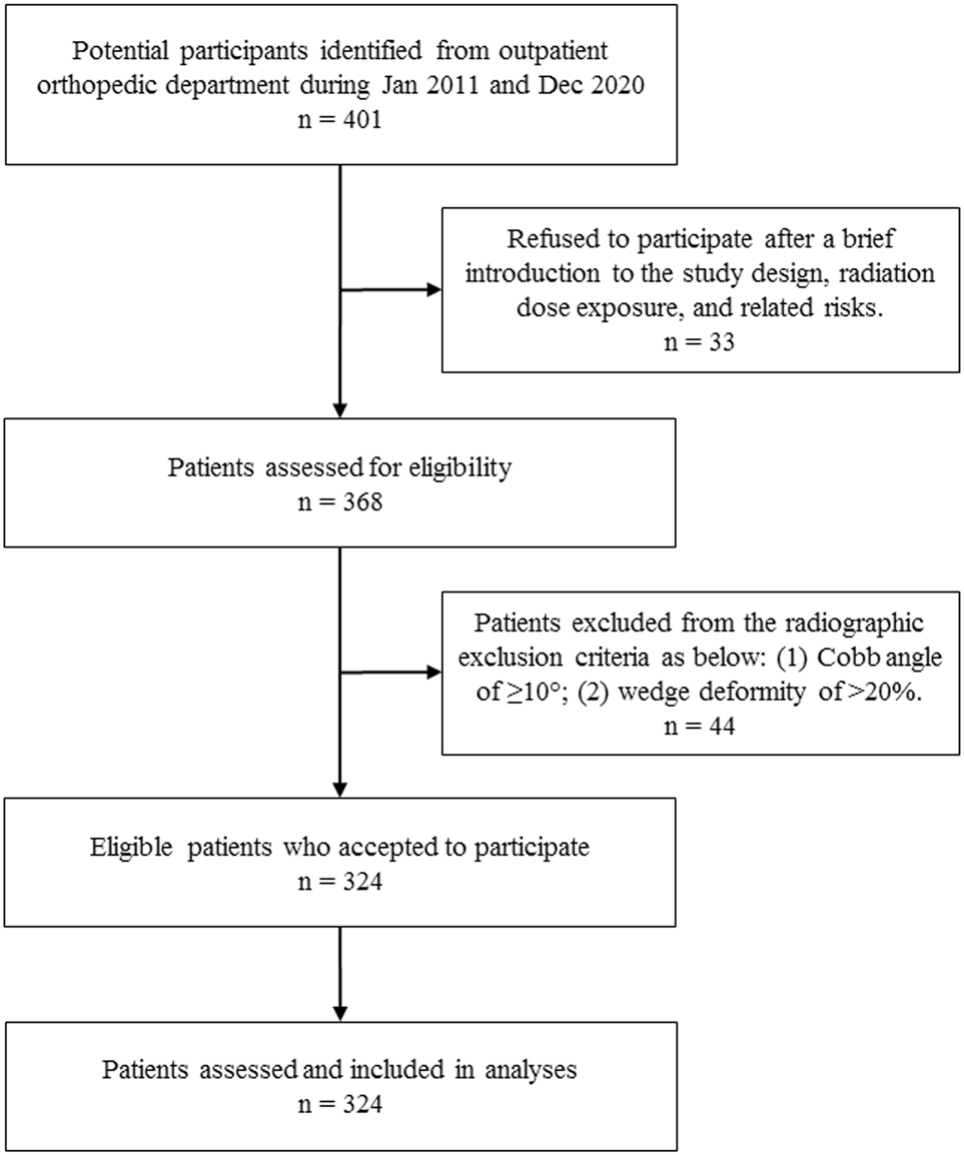
Flowchart of the study.

This study was approved by the Research Ethics Committee of Hualien Tzu Chi Hospital, Buddhist Tzu Chi Medical Foundation (IRB103-189-B). Patients who visited the hospital’s orthopedic outpatient department between January 2011 and December 2020 for upper extremity problems, who had no neck or back pain, and who could freely move their lower limbs were recruited for this study. The initial exclusion criteria were as follows: (1) a history of major surgeries of the spine, hip, or knees such as joint replacement, ligament reconstruction, fracture fixation, and spinal fusion; (2) a history of neuromuscular disorders or inflammatory arthritis; (3) recent back pain, neck pain, or lower extremity pain that affects everyday activity and requires treatment with narcotics; (4) an inability to stand without assistance; and (5) pregnancy. A total of 401 patients visiting the orthopedic outpatient department who met the inclusion criteria were recruited through convenience sampling. The effect of the complaints that the patients presented with on the standing sagittal spinopelvic alignment was small. Thirty-three patients were subsequently excluded from participating, because they did not want to undergo an X-ray examination of the whole spine. An additional 44 patients were then excluded on the basis of the following radiographic exclusion criteria: (1) a Cobb angle of ≥ 10° and (2) a wedge deformity of > 20%. Thus, a total of 324 asymptomatic patients were prospectively included; the study flowchart is illustrated in Fig. 4. Informed consent was obtained from all study participants, and the study was performed in accordance with relevant guidelines and regulations. Radiographic images were obtained under the supervision of a senior radiologist (D-WL). Standing lateral radiography of the spine was conducted with participants fully extending their knees and maintaining the fist-on-clavicle position.

**Figure 5:**
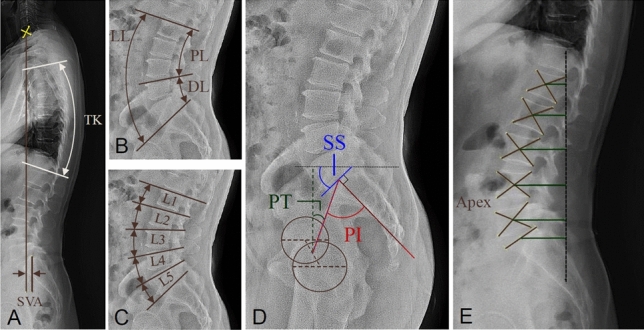
Representation of radiographic parameters. (**A**) TK denotes global thoracic kyphosis, SVA denotes sagittal vertical axis. (**B**) LL denotes lumbar lordosis, PL denotes proximal lumbar lordosis, and DL represents distal lumbar lordosis. (**C**) Segmental lordosis. (**D**) PI denotes pelvic incidence, PT represents pelvic tilt, and SS denotes sacral slope. (**E**) Lumbar apex. The maximum distance between the center of the vertebrae and the gravity line.

The following radiologic parameters were measured: thoracic kyphosis (T4–T12) and lumbosacral parameters including lumbar lordosis (L1–S1), lordosis of each lumbar segment, proximal lumbar lordosis, distal lumbar lordosis, sacral slope, pelvic incidence, pelvic tilt, the difference between pelvic incidence and lumbar lordosis, C7–S1 sagittal vertical axis, and apex vertebrae (Fig. 5). Proximal lumbar lordosis was defined as the angle between L1 and the superior endplate of L4, and distal lumbar lordosis was defined as the angle between the superior endplate of L4 and S1. Three medical students performed the radiographic measurement of the sagittal parameters, and each image was evaluated twice. The intraclass correlation coefficient (ICC) was calculated to determine interobserver and intraobserver variability. An ICC value of < 0.5 indicated poor agreement, between 0.5 and 0.75 indicated moderate agreement, between 0.75 and 0.9 indicated good agreement, and > 0.9 indicated excellent agreement^37^. Intraobserver and interobserver ICCs for the lumbopelvic sagittal parameters in this study were 0.79 (good agreement) and 0.92 (excellent agreement), suggesting the strong reliability of the measurements conducted by the three observers.

To ensure objective statistical results, we classified the greatest average number of participants into three groups according to the degrees of their pelvic incidence, in reference to Pesenti et al*.*^17^, to observe trends and analyze skeletal differences between ethnicities^13^. Participants were divided into the following similarly sized groups on the basis of pelvic incidence: G1 comprised those with a pelvic incidence of < 45°; G2 comprised those with a pelvic incidence of 45°–55°; and G3 comprised those with a pelvic incidence of > 55°. G1, G2, and G3 had 105, 111, and 108 participants, respectively.

### Statistical analysis

Statistical analyses were performed using SPSS version 22.0 (IBM, Armonk, NY, USA). We tested our data for normal distribution using the Kolmogorov–Smirnov test. Comparisons among the three pelvic incidence groups were performed using one-way analysis of variance with post hoc Bonferroni correction or the Kruskal–Wallis H test with Dunn’s post hoc test as per the normality of the data. All values are expressed as mean ± standard deviation (SD), and all error bars represent the SD of the mean. Relationships between the parameters were determined through estimating the Pearson correlation coefficient. A *P* value less than 0.05 was considered statistically significant.

## Data Availability

All data generated or analyzed during this study are included in the published article.
